# Carcinoembryonic Antigen (CEA) as a Therapeutic Target in Medullary Thyroid Cancer (MTC)

**DOI:** 10.3390/cancers18213432

**Published:** 2026-10-23

**Authors:** Kasey R. Bowyer, Marice B. Alcantara, Fang Fan, Colt A. Egelston, Paul J. Yazaki, Jeffrey Wong, Thinzar Lwin, Jack Shively, Maciej Kujawski, Krupal B. Patel

**Affiliations:** 1 Department of Clinical Science, Kaiser Permanente Bernard J. Tyson School of Medicine, Pasadena, CA 91101, USA; kasey.r.bowyer@kp.org; 2 Division of Head & Neck Surgery, Department of Surgery, City of Hope National Medical Center, Duarte, CA 91010, USA; malcantara@coh.org; 3 Department of Pathology, City of Hope National Medical Center, Duarte, CA 91010, USA; ffan@coh.org; 4 Department of Immuno-Oncology, Beckman Research Institute at City of Hope National Medical Center, Duarte, CA 91010, USA; cegelston@coh.org; 5 Department of Immunology & Theranostics, Beckman Research Institute at City of Hope National Medical Center, Duarte, CA 91010, USA; pyazaki@coh.org (P.J.Y.); jwong@coh.org (J.W.); jshively@coh.org (J.S.); mkujawski@coh.org (M.K.); 6 Department of Surgery, City of Hope National Medical Center, Duarte, CA 91010, USA; tlwin@coh.org

**Keywords:** carcinoembryonic antigen (CEA), medullary thyroid cancer (MTC), tyrosine kinase inhibitors (TKI), rearranged during transfection (RET), programmed death-ligand 1 (PD-L1), carcinoembryonic antigen-related cell adhesion molecule (CEACAM), vascular endothelial growth factor (VEGF)

## Abstract

Background/Objectives: Medullary thyroid cancer (MTC) is a rare but aggressive neuroendocrine malignancy arising from parafollicular C cells. Despite its low incidence, MTC has historically been estimated to account for 13.4% of thyroid cancer-related deaths. Survival outcomes remain limited in advanced disease, with a historical cohort reporting a 10-year survival of 21% for stage IV MTC, supporting the need for novel therapeutic approaches. Carcinoembryonic antigen (CEA), a fetal glycoprotein minimally expressed in normal adult tissues, is elevated in various malignancies and plays an established role in carcinogenesis, metastasis, and therapy resistance. In MTC, CEA is produced by neoplastic C cells alongside calcitonin, and both serum and tumor-associated CEA levels are markedly elevated relative to healthy tissue. More than half of MTC patients demonstrate elevated CEA, and intense CEA immunohistochemical staining correlates with aggressive, disseminated disease, highlighting its potential utility as both a biomarker and investigational therapeutic target in the disease. For MTC, surgery is the mainstay for locoregional disease with the intent for complete surgical resection when possible. Systemic therapy is generally reserved for structurally progressive, unresectable, symptomatic, or threatening advanced disease, with selective RET inhibitors used in RET-mutant disease and multi-kinase TKIs as additional treatment options. Although TKIs have improved progression-free survival, treatment can be limited by toxicities and the development of resistance over time. These limitations underscore the need for novel therapeutic strategies that can achieve more durable disease control and improve overall survival. Given its strong association with tumor burden, disease progression, and aggressive pathology, CEA represents a promising investigational target for therapeutic exploration in MTC. Methods: A narrative literature review was performed to evaluate carcinoembryonic antigen (CEA) as a therapeutic target and biomarker in medullary thyroid cancer (MTC). Searches were conducted in 2025 and 2026 using PubMed/MEDLINE, Embase, Cochrane Library, Scopus, Web of Science, and ClinicalTrials.gov. Conclusions: The current literature supports further investigation of CEA targeting as a therapeutic strategy in MTC. However, CEA is not yet a clinically validated therapeutic target and clinical trial data remain limited.

## Simple Summary

Medullary thyroid cancer (MTC) is a rare but aggressive neuroendocrine malignancy arising from parafollicular C cells. Despite its relative rarity, MTC has historically been estimated to account for 13.4% of thyroid cancer-related deaths. Survival outcomes are substantially worse in advanced disease, with a historical cohort of patients with MTC reporting 10-year survival of 21% for stage IV disease. This suggests novel therapeutic approaches are important in the disease. Carcinoembryonic antigen (CEA), a fetal glycoprotein minimally expressed in normal adult tissues, is elevated in various malignancies and plays an established role in carcinogenesis, metastasis, and therapy resistance. Given CEA’s strong association with tumor burden, disease progression, and aggressive pathology, CEA represents a promising investigational target for therapeutic exploration in MTC. In this review, we provide an in-depth discussion of the potential of CEA as a therapeutic target in MTC. We also provide an overview of previous and current clinical trials targeting CEA and highlight the potential and limitations of these strategies. Finally, we discuss potential novel strategies that may be utilized in the disease.

## 1. Introduction

Medullary thyroid cancer (MTC) is a rare, aggressive subtype of thyroid cancer originating from parafollicular C cells of neural crest origin, accounting for between 1 and 2% of thyroid malignancies [1,2]. Despite its rarity, MTC has historically been estimated to account for 13.4% of thyroid cancer-related cases [3]. Poor outcomes in MTC reflect multiple factors, including disease stage, tumor burden, metastatic distribution, molecular status, and biomarker kinetics. Distant metastases are present at diagnosis in approximately 10% of patients with clinically apparent MTC [4]. In a historical cohort of 899 patients with MTC, 10-year adjusted survival was 21% for stage IV disease [5]. Carcinoembryonic antigen (CEA) is a glycoprotein produced during fetal development and expressed at low levels in normal adult epithelial tissues, including the gastrointestinal tract [6]. Elevated CEA concentrations are correlated with the development of various malignancies. Indeed, CEA is associated with carcinogenesis, metastasis and treatment resistance [7]. Therefore, CEA serum concentrations are utilized to assist and guide diagnosis, prognosis, disease and treatment resistance monitoring for various cancers [8]. CEA is currently correlated with poor outcomes in lung, liver, breast and colorectal cancer [7]. In MTC, parafollicular C cells produce calcitonin and CEA, which are utilized as markers of disease progression, severity and recurrence [6]. In MTC, both serum and tumor CEA levels can be elevated in comparison to normal or surrounding tissues, and intense CEA immunohistochemical staining has been associated with aggressive and disseminated disease [6]. These associations support investigation of CEA as a therapeutic target but do not by themselves establish membrane accessibility, sufficient antigen density, homogeneous expression across lesions, or an acceptable therapeutic window. The main therapeutic option in localized or resectable MTC is complete surgical resection of the tumor and nodal metastases. Systemic therapy is generally reserved for structurally progressive, unresectable, symptomatic, or threatening advanced disease rather than biochemical marker progression alone [4]. For RET-mutant advanced MTC, selective RET inhibitors such selpercatinib are preferred, while multikinase inhibitors such as vandetanib and cabozantinib remain additional treatment options [9]. Despite some therapeutic efficacy, TKIs can be limited by toxicities and the development of acquired resistance [10,11]. While the exact mechanisms of acquired resistance are complex and multifactorial, they encompass alterations in the tumor microenvironment, drug target mutations, epigenetic mutations and changes in cell signaling pathways [12]. Collectively, these limitations suggest a need for novel therapeutic strategies to improve patient outcomes. One target that may be prudent to investigate in MTC is CEA, an established biomarker with investigational therapeutic potential in the disease.

## 2. Methods

A narrative literature review was performed to evaluate carcinoembryonic antigen (CEA) as a therapeutic target and biomarker in medullary thyroid cancer (MTC). Searches were conducted in 2025 and 2026 using PubMed/MEDLINE, Embase, Cochrane Library, Scopus, Web of Science, and ClinicalTrials.gov. The search strategy incorporated terms related to medullary thyroid cancer, carcinoembryonic antigen/CEACAM5, and CEA-directed therapeutic and diagnostic modalities, including antibody-based therapies, radioimmunotherapy, antibody-drug conjugates, CAR-based therapies, vaccines, immunotherapy, and biomarker studies. Additional search strings were used to capture the clinical, preclinical, and diagnostic/prognostic literature. Articles published in earlier years were included if relevant to the review topic. Titles and abstracts were screened, and selected studies underwent full-text review. Only full-text articles were included. Inclusion criteria comprised original research articles, clinical studies, preclinical studies, review articles, and articles published in or available in English. Exclusion criteria included conference abstracts, editorials, and articles without full-text availability.

## 3. CEA Biology and Rationale as a Therapeutic Target

### 3.1. Structure and Physiological Function of CEA/CEACAM5

**Figure 1:**
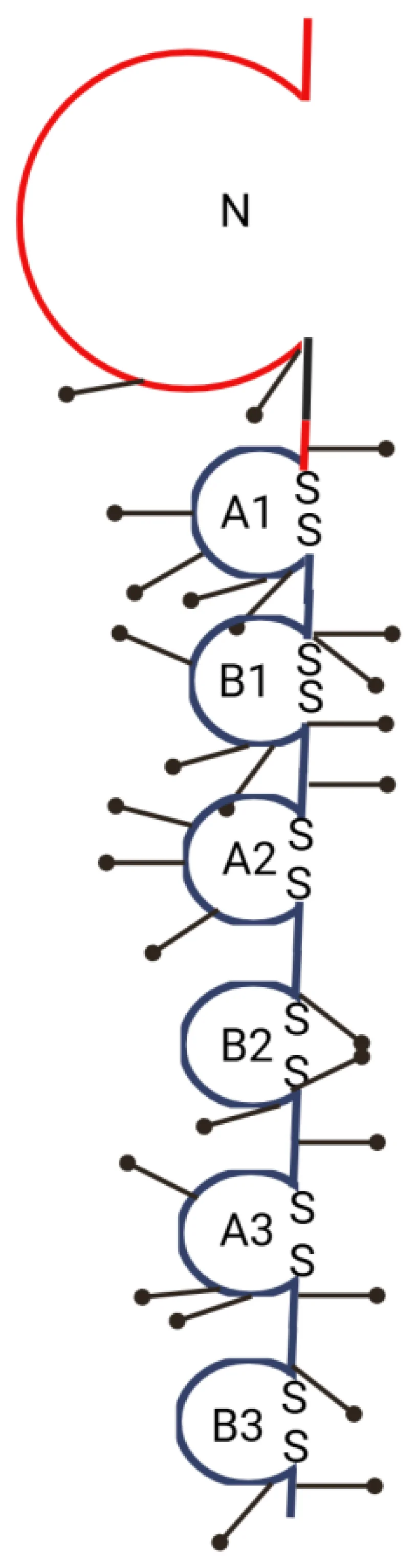
The CEA glycoprotein is approximately 150–180 kDA in size and is postulated to bear similarities to the structure of a bottle brush [20]. The primary frame is approximately 79 kDa in size, with the additional 100 kDa comprising 28 complex N-linked multi-antennary carbohydrate structures containing N-acetyl-glucosamine, mannose, galactose, fucose, and sialic acid [21]. Adapted with permission from “Carcinoembryonic antigen” P. Thomas [20]. Copyright @ 2014, Springer-Verlag Berlin Heidelberg.

The Carcinoembryonic Antigen (CEA) gene family comprises an immunoglobulin (Ig) superfamily made up of numerous glycoproteins of varying amino acid compositions and functions. The CEA family is divided into two groups: (1) carcinoembryonic antigen-related cell adhesion molecules (CEACAMs) and (2) the pregnancy-specific glycoproteins (PSGs). CEA (Figure 1) has various physiological and pathophysiological functions; for example, CEACAMs are essential during embryonic development, where cell–cell adhesion facilitates organ development. However, CEA also plays a crucial role in cancer development, with CEACAM5/CD66e currently utilized as a biomarker or therapeutic target due to its elevated levels in serum and in tumors [13]. Several other CEACAMs have also been evaluated as clinical biomarkers and therapeutic targets [14]. In this review, “CEA” refers specifically to CEACAM5 when discussing therapeutic targeting.

### 3.2. Expression Patterns in Normal Tissues vs. MTC

Under normal conditions, CEA is expressed in gastrointestinal and other adult epithelial tissues, while serum CEA concentrations in healthy individuals are generally low, typically below 5 ng/mL [7,15]. This normal epithelial expression is relevant to the potential for on-target/off-tumor toxicity with CEA-directed therapies. Comparatively, CEA is overexpressed in many tumors and present at elevated levels in the serum of patients with cancer [7,15]. As a result, CEA is currently utilized as a biomarker or therapeutic target in numerous malignancies. In a tissue microarray study of 13,725 samples obtained from 120 different tumor types, CEA was detected in 54.2% of tumors, including but not limited to colorectal adenomas and carcinomas, gastrointestinal adenocarcinomas, breast, urinary, bladder and prostate cancer [16]. CEA was significantly correlated with high-grade tumors (*p* < 0.0001) and invasive growth, suggesting an association with tumor progression and poor outcomes [16]. In another study, CEA was overexpressed in >80% of colorectal cancers [17]. In MTC, serum CEA (sCEA) is utilized as a diagnostic biomarker indicative of disease status and progression (sCEA > 30 ng/mL= extra-thyroid disease) and sCEA > 100 ng/mL = lymph node and metastatic disease [6].

### 3.3. Basis for Use as a Target (Tumor Localization, Immunogenicity)

CEA is currently utilized as a diagnostic biomarker and therapeutic tool in various malignancies due to its overexpression in tumors and relatively limited expression in normal tissues [15]. Serum CEA (sCEA) levels in MTC are currently utilized as both a biomarker and prognostic indicator, with sCEA levels over 100 ng/mL correlated with progressive and metastatic disease [6]. However, sCEA elevation, tissue immunohistochemical positivity, and prognostic association do not necessarily establish therapeutic targetability. Effective targeting requires sufficient membrane-accessible CEACAM5 on clinically relevant lesions, and expression may be heterogeneous [18]. Therefore, sCEA alone may not adequately establish tumor targetability. No validated MTC-specific CEACAM5 expression threshold currently exists. CEA-directed approaches under investigation include CAR T-cell therapy, antibody-based therapies, radioimmunotherapy, and vaccine therapies [19].

## 4. Preclinical Evidence for CEA-Targeted Therapy

Preclinical studies support CEA as an investigational therapeutic target in MTC and beyond (Table 1 and Table 2), demonstrating antitumor activity across antibody-based, radiolabeled, and cell-based immunotherapeutic treatments. Early work by Stein et al. (1999) showed that Iodine-131 (^131^I) or Yttrium-90 (^90^Y) radiolabeled anti-CEA monoclonal antibodies effectively bound CEA-positive MTC xenografts and inhibited tumor growth in mice [18]. Subsequent investigations advanced this approach through strategies that separated antibody localization from radionuclide administration. In a two-step pre-targeting system, a CEA x DTPA-indium construct followed by an iodine-131-labeled hapten induced sustained tumor growth arrest and histologic fibrosis in TT MTC xenografts, although recurrent tumors maintained CEA expression and proliferative capacity [21]. Pre-targeted delivery enhanced MTC-specific binding and achieved long-term stabilization in both CEA-expressing MTC and colorectal xenografts, reinforcing CEA’s accessibility for tumor targeting in these xenograft models [21].

Building on these results, Kraeber-Bodéré et al. (2002) combined pre-targeted CEA-radioimmunotherapy with chemotherapeutic agents paclitaxel or doxorubicin in MTC xenografts [22]. Combination with paclitaxel prolonged tumor growth suppression and produced a complete response in one animal, whereas doxorubicin failed to enhance efficacy, suggesting complementary mechanisms between microtubule stabilization and radiotherapy-induced DNA damage [22]. In a later MTC xenograft study, pretreatment with bevacizumab, an angiogenesis inhibitor, followed by anti-CEA radioimmunotherapy further improved tumor control, producing greater tumor regression and prolonged time to progression compared with radioimmunotherapy alone, without increasing measured toxicity [23]. These preclinical results suggest that CEA-directed radioimmunotherapy may be strengthened through combination strategies targeting tumor vasculature or cell-cycle regulation. Collectively, these antibody- and radionuclide-based studies support the feasibility of exploiting CEA expression for selective radiation delivery and therapeutic enhancement in preclinical MTC models.

More recently, the development of cellular therapies has expanded the preclinical landscape of CEA-targeted treatment in MTC. Erickson et al. (2022) engineered CEA-specific chimeric antigen receptor (CAR) T cells incorporating a labetuzumab-derived binding domain and optimized intracellular signaling to enhance antitumor activity [23]. These CEA-CAR T cells selectively recognized and killed CEA-positive MTC cell lines in vitro and completely eradicated MTC xenografts in vivo without signs of toxicity in the immunodeficient mouse model [19]. The findings support the preclinical feasibility of CEA as a target for CAR T-cell therapy, while the model does not reproduce normal human CEA expression or fully assess on-target/off-tumor toxicity.

Although preclinical studies in MTC remain limited, extensive work in other solid tumors provides indirect translational support for CEA-directed approaches. In colorectal and gastrointestinal cancers, multiple CEA-targeting modalities including antibody-drug conjugates (ADCs), bispecific antibodies, and vaccines have demonstrated preclinical activity. Nessler et al. (2024) showed that enhancing internalization of a CEA-targeted antibody-drug conjugate through antibody crosslinking significantly improved tumor regression and survival in colorectal carcinoma xenograft models, highlighting the importance of optimizing intracellular delivery for therapeutic potency [24]. Similarly, the bispecific CEA T-cell engager (CEA TCB) developed by Bacac et al. (2016) engineered T cells to eliminate CEA-positive tumors in vitro and in vivo, converting xenografts into highly immunogenic, PD-L1-positive lesions while maintaining antigen specificity [25]. These non-MTC findings may inform future CEA-based research in MTC but do not establish likely efficacy in MTC.

Parallel work in vaccine development has also advanced the field. CEA-based vaccines across several non-MTC cancer models, including colorectal, pancreatic, and lung cancers, have induced robust CEA-specific T-cell and antibody responses [25,26]. Viral vector-based vaccine platforms, including replication-competent vaccinia and nonreplicating canarypox (ALVAC) constructs, produced cytotoxic T-lymphocyte responses and tumor suppression. Transgenic mouse studies further showed that recombinant poxvirus-based CEA vaccines could overcome immune tolerance, reduce tumor formation, and prolong survival [26]. Together, these results provide indirect translational support for CEA-directed vaccination.

Collectively, preclinical data from non-MTC tumor models demonstrate the versatility of CEA-targeted platforms, including ADCs, bispecific antibodies, vaccines, and cellular therapies. These advances provide indirect translational support for adapting and refining CEA-directed treatments in MTC, but they do not establish clinical efficacy or likely efficacy in MTC.

**Table 2:** MTC-Specific Prospective Therapeutic Clinical Studies.

Study	Model/Approach	Key Findings	Reference
Schott, 2001	Dendritic cell vaccine with CEA and calcitonin peptides	CEA-specific immune responses; biochemical responses in 3/7 patients; one patient had complete regression of detectable liver metastases with reduction in pulmonary lesions	[27]
Salaun, 2012	Pre-targeted anti-CEA radioimmunotherapy (pRAIT)	76% disease control; one complete response; improved biomarker doubling times; hematologic toxicity in marrow cases.	[28]
Juweid, 1999	131-I-MN-14 F(ab’)2 anti-CEA radioimmunotherapy	Localized to most lesions; biochemical responses in ~50%; one partial response; repeat dosing limited by immunogenicity	[29]
NCT01856920	Yeast-CEA (GI-6207) vaccine	No significant improvement in calcitonin growth rate at 6 months; CEA-specific T-cell responses increased in 58% of treated patients; no grade 3–4 adverse events.	[30]
NCT00004048	90-Y-humanized MN-14 ± chemotherapy + stem cell rescue	Explored high-dose radioimmunotherapy; no published outcomes.	[31]

CEA, Carcinoembryonic antigen; pRAIT, pre-targeted anti-CEA radioimmunotherapy; 131-I-MN-14 F(ab’)2, a specific radiolabeled monoclonal antibody fragment; 90-Y-humanized MN-14, yttrium-90-labeled humanized MN-14 IgG, a radioimmunotherapy agent.

## 5. Clinical Evaluation of CEA-Targeted Therapies

Clinical research with CEA-targeted therapy in MTC remains limited but informative (Table 3). Early clinical testing of radioimmunotherapy using 131-I-labeled MN-14 F(ab’)2, a radiolabeled anti-CEA monoclonal antibody fragment, showed clear tumor localization and disease stabilization in most patients [29]. However, the therapy induced human anti-mouse antibodies that prevented repeat dosing and limited overall efficacy. A later prospective, multicenter, single-arm phase II trial evaluated pre-targeted anti-CEA radioimmunotherapy in progressive metastatic MTC [28]. Forty-five patients with distant metastatic disease and biochemical progression defined by rising calcitonin or CEA with doubling time <= 5 years were enrolled, and 42 completed treatment. Using Response Evaluation Criteria in Solid Tumors (RECIST), disease control, defined as objective response or stable disease lasting at least 6 months, was 76.2%, including one complete response lasting at least 40 months and 31 patients with stable disease for at least 6 months. Median progression-free and overall survival were 13.6 and 43.9 months, respectively, and 56.7% of evaluable patients had substantial lengthening of calcitonin or CEA doubling times or a prolonged biomarker decrease. Grade 3–4 hematologic toxicity occurred in 54.7% of treated patients, including leukopenia, neutropenia, thrombocytopenia, and less commonly anemia; 12 of 23 affected patients had known bone marrow involvement. Two patients with diffuse bone marrow involvement developed myelodysplastic syndrome 2 and 3.5 years after treatment. Nonhematologic toxicity included one grade 3–4 immune reaction, one grade 3 hypotension, and hepatic toxicity in seven patients, predominantly grade 1–2. These findings must be interpreted in the context of the selected, uncontrolled phase II population, and biomarker changes should be considered supportive rather than proof of clinically meaningful efficacy. Active immunization strategies have also shown that CEA-directed immunity can be induced. A dendritic cell vaccine loaded with CEA and calcitonin peptides triggered CEA-specific T-cell responses and delayed-type hypersensitivity reactions [27]. Several patients showed biochemical improvement and disease stabilization, including one with partial tumor regression. These observations demonstrate biologic activity but do not establish durable clinical benefit.

Several CEA-directed trials involving MTC (Table 2) or mixed CEA-positive tumors or advanced solid malignancies have been completed with or without publicly available MTC-specific therapeutic results. These include fowlpox-CEA-TRICOM vaccines with or without GM-CSF or dendritic-cell delivery (NCT00028496; NCT00027534; NCT00004604) and a high-dose 90-Y-MN-14 antibody with stem-cell rescue for advanced thyroid cancer (NCT00004048). The yeast-CEA GI-6207 trial in recurrent MTC (NCT01856920) is now published and did not show a significant improvement in calcitonin growth rate at six months compared with surveillance and produced no radiographic responses [30]. NCT01730638 focused on anti-CEA imaging optimization and is therefore included with diagnostic/imaging evidence in Table 4 rather than therapeutic evidence. Taken together, completed MTC studies suggest that CEA-targeted radiation can produce radiographic disease stabilization and biomarker changes in selected patients, and that vaccine strategies can induce CEA-reactive immunity, while durable objective responses remain uncommon.

No head-to-head studies compare CEA-targeted therapies with selective RET inhibitors or multikinase TKIs, and direct cross-trial comparisons are not appropriate because of differences in molecular selection, disease setting, eligibility, outcome definitions, and prior therapy. Stable disease, serum-marker reductions, and prolongation of calcitonin or CEA doubling times may indicate biologic or prognostic activity, but they are not validated measures for objective tumor response, symptom improvement, improved quality of life, or prolonged survival [27,28,29,30].

Experience in non-MTC cancers provides indirect translational support for future CEA-based research in MTC. CEA-targeted ADCs have shown disease control in heavily pretreated metastatic colorectal cancer. Labetuzumab govitecan demonstrated manageable safety and biomarker reductions, while a newer CEACAM5-exatecan conjugate achieved confirmed responses and over 50% disease control without lung or ocular toxicity, findings that may inform further optimization of payloads and linkers [32,33]. Radioimmunotherapy with 186-Re-anti-CEA produced partial responses and frequent CEA declines in colorectal cancer, demonstrating that targeted radiation delivery to CEA-positive tumors is feasible. However, bone marrow toxicity and immune reactions against the antibody limited its use, although these issues may be reduced by more modern antibody designs and pre-targeting strategies [34]. Adoptive cellular therapy confirmed that potent CEA-specific T cells can shrink colorectal metastases but also revealed severe on-target/off-tumor colitis due to recognition of normal colonic CEA expression [35]. CEA-encoding vaccine platforms can generate CEA-specific T cells, although single-agent clinical efficacy has been limited [36,37]. These findings should be considered indirect evidence and not evidence of likely efficacy in MTC.

**Table 3:** MTC Diagnostic, Prognostic, and Imaging Evidence.

Study/Trial	Model/Approach	Key Findings	Reference
Wang, 2024	Review	CEA adds value with calcitonin; higher CEA and increased CEA doubling time indicate higher-risk disease	[1]
Wells, 2015	Guideline	CEA not useful for early/stimulated diagnosis; adjunct to calcitonin for monitoring.	[4]
Wells, 1978	Clinical study	CEA often elevated but less sensitive than calcitonin; CEA not stimulant-responsive; higher with clinically evident disease.	[38]
NCCN, 2025	Guideline	Post-op surveillance uses CEA with calcitonin; doubling times guide follow-up intensity.	[36]
Trimboli, 2023	Review	Calcitonin best for diagnosis; CEA mainly prognostic in follow-up.	[37]
Hadoux, 2016	Review	CEA less sensitive/specific than calcitonin; higher/short doubling time linked to worse outcomes; rising CEA with stable CT may suggest dedifferentiation.	[9]
Wong, 2023	Pilot 64Cu-anti-CEA PET	Detected CEA-positive disease; found extra lesions in 3/5 MTC vs. CT.	[39]
Machens, 2007	Clinical study	Higher pre-op CEA correlates with advanced spread and lower chance of marker-based remission.	[40]
Prinzi, 2024	Retrospective cohort	Pre-op CEA associated with lateral neck metastases; ~17 ng/mL threshold suggested.	[41]
Barbet, 2005	Prognostic doubling-time study	Short calcitonin and CEA doubling time predict worse survival; calcitonin doubling time stronger; CEA doubling time still stratifies risk.	[42]
NCT01730638	Bispecific anti-CEA x anti-HSG PET (ImmunoTEP)	Focused on imaging optimization; no efficacy data available.	N/A

64Cu, Copper-64; CEA, Carcinoembryonic antigen; CT, computed tomography; HSG, histamine-succinyl-glycine; ImmunoTEP, the designated clinical protocol name for retargeted Immuno-PET imaging; MTC, medullary thyroid carcinoma; NCCN, National Comprehensive Cancer Network; NCT, National Clinical Trial; PET, positron emission tomography; N/A, not assessed.

**Table 4:** Indirect Non-MTC Translational Evidence.

Study/Trial	Model/Approach	Key Findings	Reference
Nessler, 2024	CEA-targeted antibody-drug conjugate (ADC) in colorectal xenografts	Faster internalization via antibody crosslinking increased tumor regression and survival without added toxicity	[24]
Bacac, 2016	Bispecific CEA T-cell engager (CEA-TCB)	Redirected T cells to kill CEA+ tumors; strong immune activation.	[25]
Berinstein, 2002	Review	Reviewed CEA-targeted vaccines; poxvirus and DC vaccines induced CEA-specific CD8+ responses and disease stabilization in early trials	[43]
Bhagat, 2023	Review	Summarized multiple CEA vaccine types (protein, DNA, RNA, viral, DC-based) showing strong immune responses and tumor inhibition in several cancers	[44]
Kujawski, 2020	Fusion protein of humanized anti-CEA with human IL-2(immunocytokine, ICK) +/− stereotactic radiation or anti-PD-1 antibody	Anti-CEA ICK improved tumor growth control and increased CD8/CD4+ T cell ratios in spleen, tumor-draining lymph nodes and tumor, suggesting improved anti-tumor responses	[45]
Hance, 2005	CEA transgenic mouse models for vaccine testing	Recombinant poxvirus CEA vaccines overcame tolerance, reduced adenoma formation, and prolonged survival without autoimmunity	[26]
Han, 2020	Review	Summarized CEACAM family; detailed antibody, BiTE, and CAR-T applications for CEA-positive solid tumors	[46]
Bos, 2008	CEA-transgenic mouse adoptive T-cell transfer	Potent T-cell activation caused tumor regression but also autoimmune colitis; demonstrated narrow safety margin	[47]
Dotan, 2017	Labetuzumab govitecan (anti-CEACAM5–SN-38 ADC)	Tumor and CEA reductions in ~40%; disease control >50%; manageable toxicity.	[32]
Kopetz, 2025	Precemtabart tocentecan (anti-CEACAM5–exatecan ADC)	7.5% partial responses; 55% disease control; favorable safety profile.	[33]
Breitz, 1992	186-Re-anti-CEA radioimmunotherapy	One partial response; frequent CEA declines; hematologic dose limits.	[34]
Parkhurst, 2011	CEA-specific TCR T-cell therapy	Tumor regression and CEA drop; severe transient colitis.	[35]
Gatti-Mays, 2020	TriAdeno vaccine (CEA/MUC1/Brachyury)	67% CEA-specific T-cell responses; no tumor regressions.	[48]
Sonpavde, 2023	CV301 (CEA/MUC1 vaccine) + atezolizumab	CEA-reactive T cells in subset; low response rate (7%).	[49]
French, 2017	Review	Highlighted clinical progress in thyroid immunotherapy; discussed CEA and vaccine strategies.	[50]
Wong, 2006	90Y-DOTA-cT84.66 anti-CEA infusion for antibody-guided radiation therapy in CEA+ cancers	Demonstrated efficacy and decreased toxicity with 90Y-DOTA-cT84.66 anti-CEA	[51]
NCT00027534	Fowlpox-CEA-TRICOM dendritic cell vaccine	Safe and feasible; no evidence of clinical benefit.	[52]
NCT00004604	Autologous dendritic cells pulsed with CEA RNA	Evaluated immune activation; no reported responses or survival data.	[53]
NCT00028496	Fowlpox-CEA(6D)/TRICOM ± sargramostim	Completed without safety issues; no objective responses reported.	[54]

186-Re, Rhenium-186; 90Y, Yttrium-90; ADC, Antibody-drug conjugate; BiTE, bispecific T-cell engager; CAR-T, chimeric antigen receptor T, CEA, carcinoembryonic antigen; CEA-TCB, carcinoembryonic antigen T-cell bispecific; cT84.66, a specific anti-CEA chimeric antibody; DC, dendritic cell; DNA, deoxyribonucleic acid; DOTA, 1,4,7,10-tetraazacyclododecane-1,4,7,10-tetraacetic acid; ICK, immunocytokine; IL-2, interleukin-2; MUC1, mucin 1; NCT, National Clinical Trial; PD-1, programmed cell death protein 1; RNA, ribonucleic acid; sargramostim, recombinant human granulocyte-macrophage colony-stimulating factor (GM-CSF)’ SN-38, the active metabolite of irinotecan; TCR, T-cell receptor; TRICOM, a triad of costimulatory molecules.

**Table 5:** Completed/Ongoing CEA- Specific Trials Without Publicly Available Therapeutic Results for other Advanced Solid Tumors.

Study/Trial	Model/Approach	Clinical Trial ID	References
Clinical Study of CEA-targeted CAR T Therapy for CEA	A study investigating safety and CEA targeted CAR T cells	NCT05415475	[55]
A Clinical Trial Targeting CEA Chimeric Antigen Receptor T (CAR T) for CEA Positive Advanced Malignant Solid Tumors	Phase I clinical trial of the drug C-13-60 (CEA-targeted CAR-T) cells in CEA positive solid tumors	NCT06043466	[56]
Actinium 225 Labeled Anti-CEA Antibody (Ac225-DOTA-M5A) for the Treatment of CEA Producing Advanced or Metastatic Cancers	Phase I clinical trial of Ac225-DOTA-M5A in patients with CEA-positive advanced colorectal cancer	NCT05204147	[57]
A study of CEA-Targeted CAR-T Therapy in Patients with CEA-Positive Advanced Solid Tumors (PBC102)	Single-arm, open-label, dose-escalating clinical study investigating safety and efficacy of CEA-targeted CAR-T cells in CEA-positive advanced tumors	NCT07250386	[58]

CEA, Carcinoembryonic antigen; CAR T, Chimeric antigen receptor (CAR) T-cell therapy; Ac225-DOTA-M5A, Actinium-225-labeled anti-CEA antibody.

## 6. CEA as a Diagnostic and Prognostic Biomarker

Serum CEA has been used for decades as a supporting biomarker to calcitonin for diagnosis and monitoring in MTC. Early work showed that basal CEA levels are frequently elevated in patients with suspected or established MTC and tend to be higher in clinically detectable MTC rather than subclinical disease, supporting CEA as a biomarker that reflects tumor burden [38]. Current guidelines incorporate CEA in addition to calcitonin in postoperative surveillance, with the intensity of follow-up informed by biomarker kinetics, particularly doubling times [1,59]. This serum biomarker role is distinct from tumoral CEACAM5 targetability, and serum CEA alone does not establish membrane-accessible CEA expression in clinically relevant lesions.

For diagnostic performance, CEA is generally less sensitive and less specific than calcitonin for detecting MTC. In early cohorts, calcitonin was more often elevated than CEA, and unlike CEA, calcitonin rose consistently with stimulation, which helps unmask subclinical disease when baseline levels are nondiagnostic, limiting CEA utility for early diagnosis or screening [4,38]. Recent reviews similarly agree that calcitonin is the most reliable diagnostic biomarker for MTC, while CEA is more informative as an adjunct during follow-up rather than as a primary diagnostic test [9,37]. Beyond serum markers, pilot anti-CEA PET immunoimaging supports that CEA can be accessible in selected lesions. In a clinical study of an anti-CEA antibody PET, scans were positive in a subset of patients, and in a small MTC subgroup the tracer identified additional lesions not seen on CT in some cases, although the dataset was small and purely diagnostic [49]. Such localization supports target accessibility in selected lesions but does not establish therapeutic efficacy, homogeneous expression, or safety.

While the literature shows CEA is limited in its diagnostic value, CEA has stronger utility as a prognostic marker, particularly when interpreted as an indicator of disease extent. Higher preoperative CEA levels correlate with greater tumor burden and metastases and increasing CEA levels have been shown to align with a reduced likelihood of tumor-marker-based cure and more advanced disease spread [1,40,41]. During follow-up, doubling time has emerged as a clinically useful prognostic measure. Short calcitonin and CEA doubling times identify high-risk patients, with calcitonin doubling time showing stronger independent prognostic performance, while CEA doubling time still provides meaningful risk stratification, particularly at very short intervals [42]. More recent reviews emphasize CEA doubling time as especially informative for identifying poor prognosis during disease monitoring, reinforcing its role in risk stratification and in guiding surveillance intensity [9,37,59].

## 7. Combination Strategies and Future Directions

Although CEA is regularly expressed in MTC and is accessible for targeting, clinical research of CEA-directed therapies has primarily demonstrated disease stabilization and biomarker effects rather than consistent tumor reduction [29,30,31]. The absence of published phase III data for CEA-targeted therapies further highlights the need to investigate strategies that strengthen and prolong treatment benefit. This rationale is reinforced by the current MTC treatment landscape, where multikinase TKIs and selective RET inhibitors are effective in advanced disease but can be limited by toxicity or resistance [40,59,60]. The current treatment landscape does not provide sustained control for all patients, creating an unmet need for well-tolerated immune-based or tumor-targeted therapies. Any CEA-directed combination strategy remains investigational and requires prospective evaluation.

Because CEA-directed therapy remains investigational, and several ongoing and clinical trials are still awaiting results (Table 5), future trials should focus on patients with structurally progressive, unresectable locoregional disease or progressive metastatic disease not amenable to curative or local therapy, including patients who have progressed on or are intolerant of genotype-appropriate systemic therapy. Resectable locoregional recurrence and oligometastatic disease should first be evaluated for surgery or other local treatment when appropriate. A practical trial algorithm should incorporate resectability, structural progression, metastatic burden, RET status, prior therapy, and tumor CEA targetability [36].

When considering CEA-directed therapeutic combination with kinase inhibitors, it is important to assess whether inhibition alters CEA expression. In an MTC preclinical model, CEA-directed CAR T cells produced complete regression of xenografts, but concurrent RET inhibition suppressed CEA expression and reduced CAR recognition and targeting capacity [19]. This antagonistic effect suggests that some combinations could downregulate surface CEA and that reassessment of antigen expression may be required to guide timing. More broadly, the thyroid cancer immunotherapy literature has proposed pairing immune modulation, including checkpoint blockade, with antiangiogenic or targeted kinase inhibitors, although this remains indirect support for MTC-specific CEA strategies [50].

Radiation-based strategies provide some of the clearest preclinical support for combination approaches in CEA-positive MTC. In xenograft models, pre-targeted CEA-directed radioimmunotherapy was enhanced by paclitaxel, prolonging tumor suppression and producing a complete response in select cases [22]. Separately, anti-VEGF pretreatment with bevacizumab improved the antitumor effect of CEA radioimmunotherapy without increasing measured toxicity, supporting vascular modulation as a method to improve delivery and therapeutic index [23]. These preclinical findings are complemented by the single-arm Phase 2 experience in progressive metastatic MTC, where anti-CEA pre-targeted radioimmunotherapy produced predominantly durable stable disease and biomarker changes [28]. Together, these findings support further study but do not establish clinical benefit for a combination regimen.

For immune-based combinations, the primary challenge is converting immune activation into consistent tumor control. In a Phase 2 study in recurrent MTC, the yeast-CEA vaccine GI-6207 induced peripheral CEA-specific T-cell responses in a substantial subset of patients and was well tolerated, but it did not significantly alter calcitonin growth kinetics at the primary six-month endpoint and produced no radiographic responses [30]. Preclinical work with a CEA T-cell bispecific antibody in non-MTC CEA-positive solid-tumor models demonstrated tumor inflammation and PD-L1 induction, providing indirect biologic rationale for studying CEA-targeted T-cell engagement with checkpoint blockade [25]. The vaccine literature similarly suggests that combinatorial approaches may enhance immunogenicity, including the use of immunostimulatory adjuvants and pairing vaccines with immune-modulating [43,44]. For example, in immune-tolerant transgenic models, adding a COX-2 inhibitor augmented vaccine effects on tumor suppression and overall survival [26]. Importantly, both transgenic models and human T-cell therapies highlight a narrow safety margin for potent CEA-directed T-cell activity due to on-target/off-target toxicity in normal gastrointestinal epithelium, emphasizing that combination intensification must be paired with safety considerations and careful patient selection [38,48].

## 8. Discussion

CEA-directed therapy remains an appealing future avenue for MTC treatment as CEA is measurable, frequently elevated, and often enriched in tumors relative to healthy surrounding tissues. However, CEA is not yet a clinically validated therapeutic target, and no head-to-head trials permit direct comparison with current standard-of-care treatments. Instead of producing consistent tumor regression, most CEA-directed approaches studied to date have primarily shown biomarker changes and disease stabilization. Stable disease, serum-marker reductions, and prolongation of calcitonin or CEA doubling times may indicate biologic activity, but they are not validated measures for objective tumor response, symptom improvement, improved quality of life, or prolonged survival. Because many of these studies were small and enrolled clinically heterogeneous populations, these modest signals are difficult to generalize and have not been sufficient to establish clinically meaningful efficacy. Overall, the data suggest CEA is a biologically relevant investigational target, but targeting CEA alone has not yet established clinical benefit.

The contemporary treatment landscape for advanced MTC provides important context for new CEA-directed approaches. Current practice is stage-driven, with surgery as the potentially curative therapy for localized or resectable disease and systemic therapy generally reserved for structurally progressive, unresectable, symptomatic, or threatening advanced disease rather than biochemical progression alone [36]. For RET-mutant advanced MTC, selective RET inhibition is preferred, while multikinase inhibitors such as cabozantinib and vandetanib remain treatment options. In LIBRETTO-531, first-line selpercatinib significantly improved progression-free survival relative to cabozantinib/vandetanib (median not reached vs. 16.8 months) and increased overall response rate (69.4% vs. 38.8%) [60]. Earlier pivotal trials established progression-free survival benefit for vandetanib and cabozantinib versus placebo [4,61,62]. These data provide treatment context [63] but are not direct comparators for historical CEA-directed studies.

The limitations observed across prior CEA-directed clinical studies point to a consistent set of challenges. First, potent T-cell-based CEA targeting faces a narrow therapeutic index because recognition of CEA on normal gastrointestinal epithelium can produce clinically significant on-target/off-tumor inflammatory colitis [35]. Soluble CEA can alter the pharmacokinetics of CEA-directed antibodies by binding circulating antibody before tumor localization, representing a potential antigen-sink effect [63]. Moreover, serum CEA concentration is not a direct measure of membrane-accessible CEACAM5 expression on tumor cells. Second, radionuclide and payload-based strategies can be limited by hematologic toxicity and marrow exposure [28]. Pre-targeted radioimmunotherapy with CEA-directed antibody-drug conjugates in other solid tumors illustrates how marrow toxicity can limit dose intensity and the ability to pursue multi-cycle regimens required for cancer control [33]. Third, earlier generations of constructs, including murine platforms, were hindered by immunogenicity that restricted repeat dosing, which is problematic for modalities that depend on iterative administrations to sustain tumor suppression.

Another shortcoming of CEA-directed therapy is inadequate and heterogeneous tumor delivery. Even in CEA-positive disease, CEACAM5/CEA-positive cells may be interspersed with antigen-low or antigen-negative compartments, and expression and lesion-level uptake can be heterogeneous, which makes it difficult for single-target ADCs or radioimmunotherapy to uniformly eliminate all malignant disease [18,39]. Antigen loss or downregulation may further reduce targetability. In an MTC preclinical model, RET inhibition reduced CEA expression and impaired CEA-directed CAR T-cell recognition [19]. Large biologics may also have limited penetration within solid tumors. In parallel, vaccine studies suggest peripheral priming often fails to produce intratumoral cytotoxicity due to immune evasion and microenvironmental suppression, so outcomes remain modest without additional immune modulation [37,49]. Together, these observations suggest that therapeutic-index constraints, antigen heterogeneity, delivery, and the tumor microenvironment all affect translation of CEA-directed therapy.

These limitations argue for a more deliberate development strategy centered on tumor targetability rather than serum CEA alone. Because no validated MTC-specific CEACAM5 threshold exists, future trials should use prespecified tissue-based criteria for membrane-accessible CEACAM5, supplemented by CEA-targeted imaging when available. Repeat biopsy may be considered when prior tissue may not reflect the current disease. Efficacy assessment should prioritize RECIST response, duration of response, progression-free survival, symptoms, patient-reported outcomes, safety, and pharmacokinetics, while CEA and calcitonin kinetics remain supportive or exploratory endpoints. Combination design should address modality-specific biological limitations rather than simply increase overall cytotoxic burden.

For CEA-directed radioimmunotherapy, heterogeneous tumor uptake and bone-marrow limited dosing argue for combinations that improve direct tumor exposure rather than simply increasing administered activity. This can be explored through delivery-priming strategies such as vascular modulation to improve penetration and make intratumoral distribution [23], pre-targeting or fractionated dosing to reduce normal-tissue radiation exposure, and radiosensitizers such as microtubule stabilizers, which prolonged tumor suppression in preclinical MTC combination trials [22]. These approaches require prospective evaluation before clinical sequencing can be recommended.

For immune-based CEA approaches, the recurring bottleneck is that peripheral immune activation does not reliably translate into sustained intratumoral cytotoxicity, supporting investigations of combinations with agents that counter adaptive resistance and microenvironmental suppression such as checkpoint blockade and other immune-enabling strategies [50]. One hypothesis is to pair CEA-directed T-cell strategies with PD-L1 blockade when adaptive resistance is induced, based on non-MTC preclinical CEA bispecific antibody data showing treatment-driven tumor inflammation and PD-L1 upregulation [25]. Vaccine experience also suggests that PD-L1 blockade alone may be insufficient without additional microenvironment modulation [49]. Other preclinical immune-enabling approaches include COX-2 inhibition, which enhanced vaccine activity in transgenic models [26].

Finally, platform engineering should be aligned to known failure modes by lowering immunogenicity to enable retreatment, since anti-drug antibody formation can reduce exposure and limit multi-cycle regimens. Potential strategies include shifting from murine to humanized constructs and de-immunizing variable regions while preserving affinity and specificity. Where repeat dosing is essential, dosing strategies can be designed to reduce immune priming, with prospective monitoring for anti-drug antibodies to inform retreatment eligibility and treatment scheduling.

Taken together, the role of CEA-directed therapy in MTC should be framed as an investigational opportunity rather than an established treatment strategy. Selective RET inhibition achieves high response rates and strong first-line progression-free survival in RET-mutant MTC [60]. Because no head-to-head trials exist, these outcomes should not be used as a direct benchmark to rank historical CEA-directed approaches. Future studies should determine whether CEA-directed therapies can provide clinical benefit in defined populations or combinations while explicitly mitigating historical barriers such as on-target/off-target toxicity, marrow dose limitation, immunogenicity, and heterogeneous tumor uptake.

A possible next step for CEA-directed therapy in MTC is a sequenced, imaging-guided approach built around anti-CEA pre-targeted radioimmunotherapy followed by genotype-matched kinase inhibition as maintenance. Among CEA-directed strategies, pre-targeted radioimmunotherapy has shown the clearest MTC-specific clinical signal, but the evidence derives from a selected, uncontrolled phase II population and its use has been limited by marrow toxicity [28]. This approach could be studied with patient selection based on tumoral CEA accessibility and marrow reserve, together with individualized dosimetry, fractionated dosing, and less immunogenic constructs. Such a sequence has not been tested clinically and should not be considered a recommended treatment pathway. Because RET pathway inhibition may reduce CEA expression and compromise targetability, the optimal timing of kinase inhibition relative to CEA-directed treatment also requires prospective study [19].

Checkpoint blockade, CEA-directed cellular therapy, and CEA vaccination likewise remain investigational combination concepts in MTC. Despite promising advances in combination strategies using immune checkpoint inhibitors and TKIs in other solid tumors [58,59,60], the typically low mutational burden of MTC may limit the potency of checkpoint inhibition in this disease [64]. CEA-directed cellular therapies, like CAR T cell therapy, face a narrow therapeutic window in solid tumors because of on-target/off-tumor toxicity and are restricted by the suppressive tumor microenvironment. The GI-6207 vaccine was well tolerated but did not significantly improve its primary calcitonin-growth endpoint and produced no radiographic responses [30]. Accordingly, no CEA-directed combination strategy can currently be preferred. Prospective studies with rigorous target selection and clinical endpoints are needed.

## 9. Conclusions

Overall, the current literature supports CEA as a biologically rational investigational therapeutic target for MTC, but CEA is not yet a clinically validated therapeutic target and evidence of clinical efficacy remains limited in the disease. Across the small and heterogeneous MTC-focused studies conducted to date, CEA-directed strategies have most consistently produced tumor localization, disease stabilization, and biomarker changes rather than consistent tumor regression and long-term durable responses. These data support further study of CEA-directed therapy but do not establish clinically meaningful efficacy.

Clinically, this argues that CEA-directed therapy should be evaluated prospectively rather than advanced as a preferred monotherapy, combination, or treatment sequence, with trial designs explicitly built to overcome known constraints in on-target/off-target toxicity, delivery, dosing, and resistance. Key gaps include defining the most defensible strategy with modern agents, improving construct properties to enable repeat dosing with an acceptable therapeutic index, and incorporating tumor-based targetability assessment and on-treatment clinical response measures to select patients and confirm appropriate target engagement early. Future MTC trials should select patients based on membrane-accessible tumor CEACAM5/CEA rather than serum CEA alone and use validated radiographic, clinical, and safety endpoints, with biomarker kinetics as supportive measures.

## Figures and Tables

**Table 1:** MTC-Specific Preclinical Therapeutic Studies.

Study	Model/Approach	Key Findings	Reference
Erickson, 2022	CEA-targeted CAR T cells	Complete regression of MTC xenografts in mice	[19]
Stein, 1999	131I/90Y-anti-CEA mAb	Significant antitumor effects in MTC xenografts	[18]
Gautherot, 1999	Bispecific Ab pre-targeting + 131-I-hapten	Tumor necrosis, reduced proliferation, persistent CEA expression	[21]
Kraeber-Bodere, 2002	Bispecific CEA Ab pre-targeting + 131-I-hapten + paclitaxel or doxorubicin	Paclitaxel improved tumor control vs. radioimmunotherapy alone; doxorubicin did not; toxicity acceptable	[22]
Salaun, 2010	131-I-anti-CEA radioimmunotherapy + bevacizumab	Improved tumor control and time to progression vs. radioimmunotherapy alone, no added toxicity	[23]

CEA, Carcinoembryonic antigen; CAR-T, Chimeric antigen receptor (CAR) T-cell; MTC, medullary thyroid carcinoma; Ab, antibody.

## Data Availability

No new data were created or analyzed in this study. Data sharing is not applicable to this article.

## Abbreviations

The following abbreviations are used in this manuscript

CEA	Carcinoembryonic Antigen
MTC	Medullary Thyroid Carcinoma
TKI	Tyrosine Kinase Inhibitors
RET	Rearranged During Transfection
PD-L1	Programmed death-ligand 1
CEACAM	Carcinoembryonic Antigen-Related Cell Adhesion Molecule
VEGF	Vascular Endothelial Growth Factor
